# Supplementary material for: The Non-Recurrent Laryngeal Nerve: a meta-analysis and clinical considerations
Source: PeerJ. 2017 Mar 21;5:e3012. doi: 10.7717/peerj.3012 (PMC5363258; doi:10.7717/peerj.3012)
Supplement: Table S1 [file peerj-05-3012-s001.docx]

**Supporting Table 1 - PubMed Search Strategy**

| 1 | ((("recurrent laryngeal nerve"[Title/Abstract]) OR "nervus laryngeus recurrens"[Title/Abstract]) OR "inferior laryngeal nerve"[Title/Abstract]) OR "inferior thyroid artery"[Title/Abstract] |
| --- | --- |
| 2 | (((((("anatomy"[Title/Abstract]) OR "variation"[Title/Abstract]) OR "anomaly"[Title/Abstract]) OR "course"[Title/Abstract]) OR "relationship"[Title/Abstract]) OR "branching"[Title/Abstract]) OR "division"[Title/Abstract] |
| 3 | 1 AND 2 |
| 4 | ("recurrent laryngeal nerve/anatomy and histology"[MeSH Major Topic]) |
| 5 | "non recurrent laryngeal nerve"[Title/Abstract] |
| 6 | “Zuckerkandl's Tubercle” |
| 7 | “Galen’s anastomosis” OR “Arytenoid plexus” OR “Cricoid anastomosis” OR “Thyroarytenoid anastomosis” OR “cricothyroid anastomosis” OR “human communicating nerve” |
| 8 | 3 OR 4 OR 5 OR 6 OR 7 |
