# Supplementary material for: The Non-Recurrent Laryngeal Nerve: a meta-analysis and clinical considerations
Source: PeerJ. 2017 Mar 21;5:e3012. doi: 10.7717/peerj.3012 (PMC5363258; doi:10.7717/peerj.3012)
Supplement: Table S3 [file peerj-05-3012-s003.pdf]

Study ID	Region	Country	Type of Study	n= Right RLNs examined	# of NRLN R
Ardito 2004	Europe	Italy	Intraoperative	1342	5
Asgharpour 2012	Europe	England	Cadaveric	143	1
Barczynski 2015	Europe	Poland	Intraoperative	1250	9
Beneragama 2006	Oceania	Australia	Intraoperative	114	1
Benouaich 2012	Europe	France	Cadaveric	10	0
Bula 2015	Europe	Poland	Intraoperative	1710	4
Cai 2013	Asia	China	Intraoperative	783	4
Campos 2000	South America	Brazil	Cadaveric	71	0
Chiang 2012	Asia	Taiwan	Intraoperative	310	4
Dolezel 2015	Europe	Czech Republic	Intraoperative	725	4
Donatini 2013	Europe	Italy	Intraoperative	402	11
Flament 1983	Europe	France	Cadaveric	100	2
Freschi 1994	Europe	Italy	Intraoperative	42	0
Han 2015	Asia	China	Intraoperative	1056	6
Henry 1988	Europe	France	Intraoperative	4921	31
Hermans 2003	Europe	Belgium	Intraoperative	484	1
Hong 2014	Asia	Korea	Intraoperative	2187	15
Hunt 1968	Oceania	Australia	Cadaveric	77	1
Iacobone 2015	Europe	Italy	Intraoperative	1477	17
Kaisha 2011	Africa	Kenya	Cadaveric	73	1
Kandil 2011	North America	USA	Intraoperative	162	0
Lee 2009	Asia	Korea	Cadaveric	70	0
Lekacos 1992	Europe	Greece	Intraoperative	109	1
Makay 2008	Europe	Turkey	Intraoperative	250	0
Maranillo 2008	Europe	England	Cadaveric	137	1
Menck 1990	Europe	Germany	Cadaveric	101	0
Monfared 2002	North America	USA	Cadaveric	21	1
Moreau 1998	Europe	France	Cadaveric	17	0
Ngo Nyeki 2015	Africa	Cameroon	Intraoperative	32	0

Page 2003	Europe	France	Intraoperative	205	0
Papadatos 1978	Europe	Switzerland	Cadaveric	239	2
Pradeep 2012	Asia	India	Intraoperative	324	1
Proye 1982	Europe	France	Intraoperative	2490	15
Raffaelli 2000	Europe	France	Intraoperative	656	3
Reed 1943	North America	USA	Cadaveric	253	3
Sasou 1998	Asia	Japan	Intraoperative	367	0
Satoh 2013	Asia	Japan	Intraoperative	1561	11
Sciumè 2005	Europe	Italy	Intraoperative	263	2
Serpell 2009	Oceania	Australia	Intraoperative	432	1
Shao 2010	Asia	China	Intraoperative	1988	12
Shindo 2005	North America	USA	Intraoperative	149	1
Silva 2013	South America	Brazil	Cadaveric	106	1
Skandalakis 1976	North America	USA	Cadaveric	102	1
Sparta 2004	Europe	France	Intraoperative	274	2
Stewart 1972	North America	USA	Intraoperative	1776	6
Sturniolo 1999	Europe	Italy	Intraoperative	141	0
Sunanda 2010	Asia	Sri Lanka	Intraoperative	24	1
Tang 2012	Asia	China	Cadaveric	80	2
Wade 1955	Europe	England	Cadaveric	100	4
Wang 2011	Asia	China	Intraoperative	290	9
Watanabe 2001	Asia	Japan	Imaging (CT)	594	6
Watanabe 2016	Asia	Japan	Intraoperative	730	4
Yang 2014	Asia	China	Intraoperative	2251	28

Study ID	# of NRLN R	NRLN with arteria lusoria
Asgharpour 2012	1	1
Bula 2015	4	0
Cai 2013	4	4
Chiang 2012	4	4
Dolezel 2015	4	4
Flament 1983	2	2
Henry 1988	31	31
Hermans 2003	1	0
Hong 2014	15	14
Hunt 1968	1	1
Maranillo 2008	1	1
Monfared 2002	1	1
Papadatos 1978	2	2
Raffaelli 2000	3	3
Reed 1943	3	3
Satoh 2013	11	10
Skandalakis 197	1	1
Wang 2011	9	6
Watanabe 2001	6	6
Watanabe 2016	4	4
Yang 2014	28	28

Study ID	# of NRLN R	Type A (origin at or above LTJ)	Tybe B (origin below LTJ)
Asgharpour 2012	1	1	0
Bula 2015	4	4	0
Cai 2013	4	1	3
Chiang 2012	4	1	3
Dolezel 2015	4	0	4
Henry 1988	33	7	26
Hong 2014	15	12	3
Kaisha 2011	1	1	0
Maranillo 2008	1	0	1
Monfared 2002	1	1	0
Papadatos 1978	2	1	1
Reed 1943	3	3	0
Stewart 1972	6	4	2
Tang 2012	2	2	0
